# Supplementary material for: Causal interplay between lactose intolerance and gut microbiota: a combined bidirectional Mendelian randomization and in vivo validation study
Source: Front Nutr. 2026 Jun 1;13:1803337. doi: 10.3389/fnut.2026.1803337 (PMC13265576; doi:10.3389/fnut.2026.1803337)
Supplement: Supplementary file 1 [file Data_Sheet_1.zip › supplementary materials/Forward/leave one out cross validation/ebi-a-GCST90027571.finngen_R12_E4_LACTONAS.pdf]

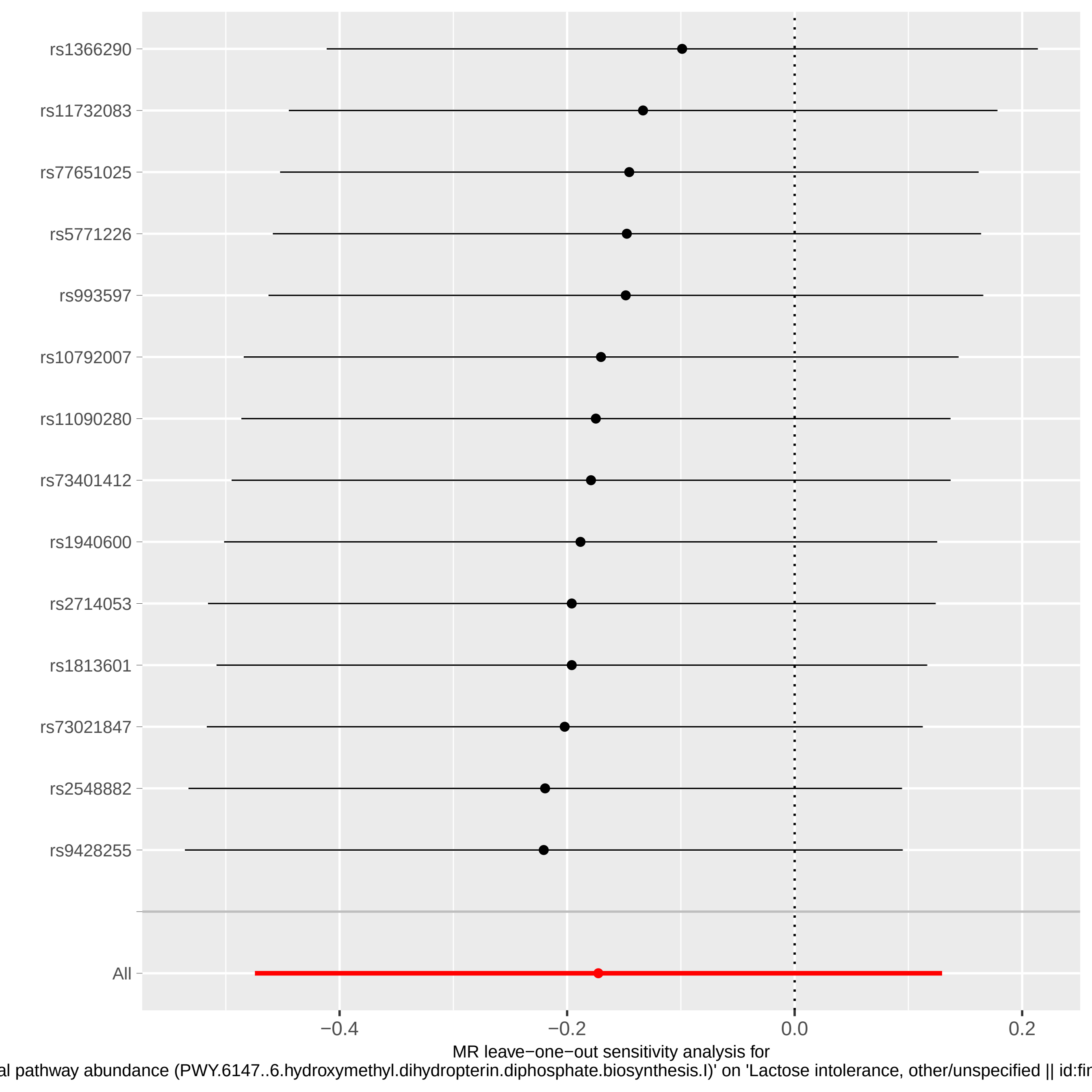
rs1366290
rs11732083
rs77651025
rs5771226
rs993597
rs10792007
rs11090280
rs73401412
rs1940600
rs2714053
rs1813601
rs73021847
rs2548882
rs9428255
All
−0.4
−0.2
0.0
0.2
MR leave−one−out sensitivity analysis for
al pathway abundance (PWY.6147..6.hydroxymethyl.dihydropterin.diphosphate.biosynthesis.I)' on 'Lactose intolerance, other/unspecified || id:fir
